# Respiratory Impairment Predicts Response to IL-1 and IL-6 Blockade in COVID-19 Patients With Severe Pneumonia and Hyper-Inflammation

**DOI:** 10.3389/fimmu.2021.675678

**Published:** 2021-04-29

**Authors:** Emanuel Della-Torre, Marco Lanzillotta, Corrado Campochiaro, Giulio Cavalli, Giacomo De Luca, Alessandro Tomelleri, Nicola Boffini, Rebecca De Lorenzo, Annalisa Ruggeri, Patrizia Rovere-Querini, Antonella Castagna, Giovanni Landoni, Moreno Tresoldi, Fabio Ciceri, Alberto Zangrillo, Lorenzo Dagna

**Affiliations:** ^1^ Università Vita-Salute San Raffaele, IRCCS San Raffaele Scientific Institute, Milan, Italy; ^2^ Unit of Immunology, Rheumatology, Allergy and Rare Diseases, IRCCS San Raffaele Scientific Institute, Milan, Italy; ^3^ Hematology and Bone Marrow Transplant Unit, IRCCS San Raffaele Scientific Institute, Milan, Italy; ^4^ Division of Immunology, Transplantation and Infectious Diseases, IRCCS San Raffaele Scientific Institute, Milan, Italy; ^5^ Department of Infectious Diseases, IRCCS San Raffaele Scientific Institute, Milan, Italy; ^6^ Department of Anesthesia and Intensive Care, IRCCS San Raffaele Scientific Institute, Milan, Italy; ^7^ General Medicine and Advanced Care Unit, IRCCS San Raffaele Scientific Institute, Milan, Italy

**Keywords:** COVID-19, SARS-CoV-2, interleukin-6, interleukin-1, sarilumab, anakinra, tocilizumab

## Abstract

**Background:**

Restraining maladaptive inflammation is considered a rationale strategy to treat severe *coronavirus disease-19* (COVID-19) but available studies with selective inhibitors of pro-inflammatory cytokines have not provided unequivocal evidence of survival advantage. Late administration is commonly regarded as a major cause of treatment failure but the optimal timing for anti-cytokine therapy initiation in COVID-19 patients has never been clearly established.

**Objectives:**

To identify a window of therapeutic opportunity for maximizing the efficacy of interleukin (IL)-1 and IL-6 blockade in COVID-19.

**Methods:**

Survival at the longest available follow-up was assessed in severe hyper-inflamed COVID-19 patients treated with anakinra, tocilizumab, sarilumab, or standard of care, stratified according to respiratory impairment at the time of treatment initiation.

**Results:**

107 patients treated with biologics and 103 contemporary patients treated with standard of care were studied. After a median of 106 days of follow-up (range 3-186), treatment with biologics was associated with a significantly higher survival rate compared to standard therapy when initiated in patients with a PaO_2_/FiO_2_ ≥ 100 mmHg (p < 0.001). Anakinra reduced mortality also in patients with PaO_2_/FiO_2_ < 100 mmHg (p = 0.04).

**Conclusions:**

IL-1 and IL-6 blocking therapies are more likely to provide survival advantage in hyper-inflamed COVID-19 patients when initiated before the establishment of severe respiratory failure.

## Introduction

Life-threatening *coronavirus disease-19* (COVID-19) is sustained by a maladaptive inflammatory response induced by the *Severe Acute Respiratory Syndrome Coronavirus 2* (Sars-CoV-2) (1, 2). This hyper-inflammatory response resembles the “cytokine storm” observed during macrophage-activation syndrome and is characterized by excessive release of pro-inflammatory cytokines including interleukin (IL)-1, IL-6, tumor necrosis factor, and granulocyte–macrophage colony stimulating factor, among others (1, 2).

Based on this hyper-inflammatory pathogenic background, targeting upstream molecules in the inflammatory cascade such as IL-1 and IL-6 soon appeared as a promising therapeutic strategy to contrast the progression of severe COVID-19, and high hopes were placed on the IL-1 and IL-6 blocking agents anakinra, tocilizumab, and sarilumab (3–10). In particular, anakinra is a recombinant replica of the IL-1 receptor antagonist that binds to soluble IL-1α and IL-1β preventing their pro-inflammatory activity (10). Tocilizumab and sarilumab, on the other hand, are humanized monoclonal antibodies that block IL-6-mediated signal transduction by targeting both the membrane and soluble forms of the IL-6 receptor (3). Yet, despite great expectations and encouraging outcomes from preliminary observational cohorts, tocilizumab and sarilumab lately failed to demonstrate an impact on disease mortality in open-label and randomized clinical trials (3–8). Similarly, recently published controlled investigations with anakinra also returned an overall unclear survival benefit compared to supportive therapies (10–12). Of note, non-response to IL-1 and IL-6 blocking therapies has been commonly attributed to late administration of these agents at advanced disease stage but the optimal timing of anti-inflammatory treatment initiation in COVID-19 has never been clearly established.

During the first COVID-19 surge that stroke Lombardy region (Italy) in the spring of 2020, our Institute hospitalized 954 patients and rapidly set in place contemporary open label observational studies of anakinra, sarilumab and tocilizumab to treat severe hyper-inflamed COVID-19 patients fulfilling common sets of inclusion criteria (3, 4, 9, 10, 13, 14). This unique experience on a homogeneous population cohort now provides an unprecedented occasion to address clinical and serological variables associated with COVID-19 response to anti-cytokine therapies, and to identify a “window of therapeutic opportunity” for maximizing the efficacy of IL-1 and IL-6 blockade in these patients.

## Methods

### Study Population and Eligibility Criteria

This study was conducted from February 25^th^ 2020, through May 20^th^ 2020 at San Raffaele Hospital (Milan, Italy) during the first COVID-19 outbreak in Lombardy region. Hospitalized patients were recruited in an Institutional observational protocol (COVIDBioB Study, Ethical Committee approval no. 34/int/2020, ClinicalTrials.gov NCT04318366) and gave written informed consent to compassionate off-label use of anakinra, sarilumab, and tocilizumab. Patients eligible to anti-cytokine therapy were required to have confirmed SARS-CoV-2 infection by reverse-transcriptase polymerase-chain-reaction on nasal-pharyngeal swab and radiologically documented bilateral pneumonia. In addition patients were required to have severe COVID-19 as defined by a partial pressure of arterial oxygen/fraction of inspired oxygen (PaO2/FiO2) ratio ≤ 300 mmHg on high flow supplemental oxygen, and a hyper-inflamed phenotype as defined by an elevation of lactate dehydrogenase (LDH) above the upper limit of normal (ULN), and at least one of the following: C-reactive protein (CRP) ≥ 100 mg/L; IL-6 ≥ 40 pg/ml; or ferritin ≥ 900 ng/ml. Patients hospitalized for more than 14 days, on concomitant or previous immunosuppressive agents, or mechanically ventilated were excluded. Patients with uncontrolled systemic infections, total neutrophil count < 1500/mm3, serum levels of alanine aminotransferase and aspartate aminotransferase more than five times the ULN, diverticulitis/diverticulosis, and pregnant women were also excluded. Contemporary patients fulfilling the same inclusion/exclusion criteria and matched for age, comorbidities, inflammatory markers, and respiratory parameters were identified and used as a comparison group. These patients did not receive anti-cytokine therapy due to drug unavailability at the time of hospital admission or lack of consent. Patients admitted to intensive care units (ICU) or dying within 24 hours from criteria fulfillment were excluded to avoid potential biases favoring one or the other group.

### Study Design and Treatments

The main objectives of the study were to (i) describe the long-term survival of patients treated with anti-cytokine biologic agents compared to patients treated with local standard of care; (ii) to compare the efficacy of different biologic agents in terms of mortality rate; (iii) to identify possible predictors of response to anti-cytokine therapies. Treatment with biologic drugs was initiated in addition to local standard of care on a compassionate indication within 24 hours from the fulfilment of inclusion criteria outside ICU. Anakinra was administered intravenously at a dose of 5 mg/Kg twice daily (total daily dose: 10 mg/kg) until clinical benefit, defined as sustained improvement of respiratory parameters. Tocilizumab was administered intravenously as a single dose of 400 mg, which was repeated after 24 hours if the respiratory function further worsened. Sarilumab was administered intravenously as a single dose of 400 mg. All patients received oral therapy with lopinavir/ritonavir, hydroxychloroquine and a course of azithromycin as per local institutional standard of care at the time of admission (see next section). Supportive therapies with supplemental oxygen and/or non-invasive ventilation (NIV) with continuous positive airway pressure (CPAP, with a positive end expiratory pressure [PEEP] of 10 cm of H2O) were provided at the discretion of the caring clinicians. Patients were prospectively followed-up with daily data collection into an electronic case report form until death or discharge. Patients transferred to rehabilitation were also followed-up through clinical records. There has been no patient or public involvement in the conception, design, and conduction of the present study.

### Standard of Care Treatment

*Antiviral therapy*: in the absence of specific contraindications, all patient included in this study received: Hydroxychloroquine 200 mg BD orally and Lopinavir/Ritonavir, 400/100 mg BD orally. *Steroids*: glucocorticoids were not part of the standard of care and were not used to treat enrolled patients, although in some circumstances they have been used for relieving bronchospasm or for treating hypersensitivity drug reactions. Being on other immunosuppressive agents was considered an exclusion criteria. *Antibiotic therapy*: all patients received an initial empiric antibiotic coverage for community acquired/hospital acquired pneumonia based on either daily intravenous Ceftriaxone 2 g or Azithromycin 500 mg. For patients with negative cultures and decreasing inflammatory markers ceftriaxone was discontinued after 6 days of therapy.

### Statistical Analysis

Statistical analysis was performed using Prism software 8.0 (GraphPad Software, La Jolla, CA, USA) and SPSS v26 (SPSS, Chicago, IL-USA). Continuous variables are reported as medians and interquartile ranges. Categorical variables are reported as numbers and percentages. Wilcoxon rank-sum tests were applied to continuous variables and two-tailed Fisher’s exact tests were used for categorical variables. Pearson chi-square was employed while analysis frequencies of more than two groups. Survival analysis was performed with the Kaplan-Meier plots, and log-rank test was used to compare survival curves. Hazard ratio (HR) of survival were estimated using Cox regression models with backward selection method. Results of the Cox regression model are presented as HR with 95% confidence interval. Variables with a p-value < 0.2 on univariate analysis were subsequently entered into the final multivariate model. P-values <0.05 were considered statistically significant.

## Results

### Baseline Characteristics of the Patients’ Cohorts

210 subjects fulfilling the inclusion criteria were included. 107 (50.9%) patients were treated with biologic agents in addition to local standard of care: 52 (48.6%) with anakinra; 30 (28%) with tocilizumab; and 25 (23.4%) with sarilumab (**Tables 1**–**4**). 103 (49.1%) concomitantly hospitalized patients matched according to the inclusion criteria and treated with local standard of care were used as comparators (**Table 1**). All patients were on high-flow oxygen supplementation or on NIV as per clinical judgement due to acute respiratory distress syndrome (ARDS) (PaO2/FiO2 ratio <300 with a PEEP ≥5 cm H2O).

**Table 1:** Clinical and serological features of the patient cohort.

	Biologics(n=107)	Standard of care(n=103)	p value
**Age (years)**	61 (54-72)	62 (57-72)	0.15
**Male sex, n (%)**	67 (62.6%)	57 (55%)	0.33
**Comorbidities**			
HBP	39 (35%)	44 (43%)	0.49
CAD	11 (10%)	18 (17%)	0.17
Type 2 diabetes	17 (16%)	21 (20%)	0.38
COPD	4 (4%)	7 (7%)	0.36
Cancer	5 (5%)	8 (8%)	0.58
CRF	7 (7%)	7 (7%)	0.99
**PaO2/FiO2 ratio (mmHg)**	91 (75-132)	105 (79-188)	0.1
**Laboratory values**			
LAD (125-220 IU/L)	453 (377-579)	474 (383-606)	0.38
CRP (< 6 mg/L)	154 (111-219)	167.6 (124.8-231)	0.11
Ferritin (30-400 ng/mL)	1542 (1009-3030)	2260 (1237-3450)	0.11
IL6 (<7 pg/mL)	57 (37-117)	68.9 (34.65-162)	0.63

Results are reported as median (interquartile range) unless specified; statistically significant p value < 0.05. HBP, high blood pressure; CAD, coronary artery disease; COPD, chronic obstructive pulmonary disease; CRF, chronic renal failure; LAD, lactate dehydrogenase; CRP, C-reactive protein; IL-6, interleukin-6.

**Table 2:** Clinical and serological features of patients treated with anakinra.

	Anakinra (n=52)	Standard of care(n=88)	p value	PaO2/FiO2 ratio ≥ 100 mmHg			PaO2/FiO2 ratio < 100 mmHg		
				Anakinra(n=16)	Standard of care(n=43)	p value	Anakinra(n=36)	Standard of care(n=45)	p value
**Age (years)**	63 (55-74)	62 (57-73)		60 (51-71)	61 (55-72)	0.77	64 (56-74)	63 (58-73)	0.9
**Male sex, n (%)**	33 (63%)	50 (57%)	0.59	13 (81%)	31 (72%)	0.74	20 (56%)	19 (42%)	0.27
**Comorbidities**									
HBP	22 (42%)	39 (44%)	0.86	4 (25%)	17 (40%)	0.37	18 (50%)	22 (49%)	0.99
CAD	6 (11%)	16 (18%)	0.34	0 (0%)	8 (19%)	0.09	6 (16%)	8 (18%)	0.99
Type 2 diabetes	12 (23%)	18 (21%)	0.83	1 (6%)	8 (19%)	0.42	11 (30%)	10 (22%)	0.45
COPD	3 (6%)	7 (8%)	0.74	0 (0%)	3 (7%)	0.56	3 (8%)	4 (9%)	0.99
Cancer	2 (4%)	8 (9%)	0.32	1 (6%)	7 (16%)	0.43	1 (3%)	1 (2%)	0.99
CRF	4 (8%)	4 (5%)	0.47	0 (0%)	1 (2%)	0.99	4 (11%)	3 (7%)	0.69
**PaO2/FiO2 ratio (mmHg)**	81 (70-113)	96 (72-143)	0.09	143 (117-181)	143 (122-189)	0.84	77 (67-85)	73 (61-86)	0.74
**Laboratory values**									
LAD (125-220 IU/L)	458 (372-580)	510 (393-630)	0.21	392 (286-551)	428 (383-542)	0.25	499 (389-591)	565 (436-726)	0.08
CRP (< 6 mg/L)	157 (120-224)	171 (122-232)	0.36	130 (102-162)	153 (119-199)	0.12	165 (128-240)	202 (135-238.	0.65
Ferritin (30-400 ng/mL)	1547 (937-3521)	2347 (1242-3549)	0.17	1773 (912-3347)	2486 (1291-3436)	0.4	1462 (929-3847)	1717 (1198-3871)	0.33
IL6 (<7 pg/mL)	48 (27-99)	65 (37-172)	0.63	75 (69-82)	47 (26-153)	0.22	59 (42-831)	105 (66-287)	0.41

Results are reported as median (interquartile range) unless specified; statistically significant p value < 0.05. HBP, high blood pressure; CAD, coronary artery disease; COPD, chronic obstructive pulmonary disease; CRF, chronic renal failure; LAD, lactate dehydrogenase; CRP, C-reactive protein; IL-6, interleukin-6.

**Table 3:** Clinical and serological features of patients treated with sarilumab.

	Sarilumab (n=25)	Standard of care(n=68)	p value	PaO2/FiO2 ratio ≥ 100 mmHg			PaO2/FiO2 ratio < 100 mmHg		
				Sarilumab (n=8)	Standard of care(n=33)	p value	Sarilumab (n=17)	Standard of care(n=35)	p value
**Age (years)**	56 (50-62)	61 (55-67)	0.07	56 (52-62)	60 (52-64)	0.74	56 (48-60)	61 (57-64)	0.07
**Male sex, n (%)**	16 (64%)	37 (54%)	0.48	6 (75%)	23 (70%)	0.99	10 (59%)	14 (40%)	0.24
**Comorbidities**									
HBP	5 (20%)	27 (39%)	0.09	1 (12%)	10 (30%)	0.41	4 (23%)	17 (49%)	0.13
CAD	2 (8%)	8 (12%)	0.72	1 (12%)	2 (6%)	0.49	1 (6%)	6 (17%)	0.4
Type 2 diabetes	1 (4%)	14 (21%)	0.07	0 (0%)	6 (18%)	0.32	1 (6%)	8 (23%)	0.24
COPD	0 (0%)	5 (7%)	0.32	0 (0%)	1 (3%)	0.99	0 (0%)	4 (11%)	0.29
Cancer	1 (4%)	6 (9%)	0.67	0 (0%)	6 (18%)	0.32	1 (6%)	0 (0%)	0.33
CRF	0 (0%)	2 (3%)	0.9	0 (0%)	0 (0%)	0.99	0 (0%)	2 (6%)	0.99
**PaO2/FiO2 ratio (mmHg)**	87 (74-123)	96 (71-146)	0.38	138 (122-184)	148 (116-187)	0.98	81 (69-87)	72 (61-89)	0.48
**Laboratory values**									
LAD (125-220 IU/L)	477 (420-591)	522 (403-632)	0.47	444 (327-489)	459 (386-541)	0.46	488 (438-602)	585 (447-726)	0.21
CRP (< 6 mg/L)	132 (102-219)	176 (122-230)	0.23	143 (112-179)	153 (118-192)	0.58	122 (100-241)	202 (132-238)	0.24
Ferritin (30-400 ng/mL)	1890 (1028-3050)	2486 (1237-3500)	0.36	1098 (876-1550)	1876 (1217-3450)	0.07	2611 (1410-3883)	1482 (1077-3720)	0.7
IL6 (<7 pg/mL)	60 (38-129)	51 (28-143)	0.41	88 (43-164)	42 (26-170)	0.27	60 (36-127)	99 (66-270)	0.1

Results are reported as median (interquartile range) unless specified; statistically significant p value < 0.05. HBP, high blood pressure; CAD, coronary artery disease; COPD, chronic obstructive pulmonary disease; CRF, chronic renal failure; LAD, lactate dehydrogenase; CRP, C-reactive protein; IL-6, interleukin-6.

**Table 4:** Clinical and serological features of patients treated with tocilizumab.

	Tocilizumab(n=30)	Standard of care(n=103)	p value	PaO2/FiO2 ratio ≥ 100 mmHg			PaO2/FiO2 ratio < 100 mmHg		
				Tocilizumab(n=18)	Standard of care(n=59)	p value	Tocilizumab(n=12)	Standard of care(n=44)	p value
**Age (years)**	62.5 (53-74)	62 (57-72)	0.86	58 (51-73)	61 (55-71)	0.56	72 (59-74)	63 (58-73)	0.56
**Male sex, n (%)**	18 (60%)	57 (55%)	0.68	12 (67%)	38 (64%)	0.99	6 (50%)	19 (43%)	0.75
**Comorbidities**									
HBP	12 (40%)	44 (43%)	0.83	7 (39%)	22 (37%)	0.99	5 (42%)	23 (52%)	0.75
CAD	3 (10%)	18 (17%)	0.4	0 (0%)	10 (17%)	0.1	3 (25%)	8 (18%)	0.69
Type 2 diabetes	4 (13%)	21 (20%)	0.44	2 (11%)	10 (17%)	0.72	2 (17%)	11 (25%)	0.71
COPD	1 (3%)	7 (7%)	0.68	0 (0%)	3 (5%)	0.99	1 (8%)	4 (9%)	0.99
Cancer	2 (6%)	8 (8%)	0.99	1 (5%)	7 (12%)	0.67	1 (8%)	1 (2%)	0.39
CRF	3 (10%)	7 (7%)	0.69	0 (0%)	3 (5%)	0.99	3 (25%)	4 (9%)	0.16
**PaO2/FiO2 ratio (mmHg)**	111 (84-181)	121 (91-221)	0.83	176 (117-187)	172 (127-229)	0.35	81 (70-86)	73 (61-86)	0.26
**Laboratory values**									
LAD (125-220 IU/L)	441 (358-552)	474 (383-606)	0.39	443 (354-552)	415 (377-540)	0.66	438 (362-583)	565 (436-726)	0.08
CRP (< 6 mg/L)	143 (92-212)	167.6 (124-231)	0.17	124 (68-187)	153 (123-192)	0.09	186 (139-234)	202.6 (135-238)	0.77
Ferritin (30-400 ng/mL)	1400 (1054-2654)	2260 (1237-3450)	0.1	1433 (1104-3079)	2326 (1254-3215)	0.47	1228 (789-2573)	1717 (1198-3871)	0.07
IL6 (<7 pg/mL)	69 (50-456)	68.9 (34-162)	0.73	56 (29-229)	51 (26-116)	0.71	45 (24-52)	105 (66-286)	0.11

Results are reported as median (interquartile range) unless specified; statistically significant p value < 0.05. HBP, high blood pressure; CAD, coronary artery disease; COPD, chronic obstructive pulmonary disease; CRF, chronic renal failure; LAD, lactate dehydrogenase; CRP, C-reactive protein; IL-6, interleukin-6.

### Predictors of Survival

**Figure 1:**
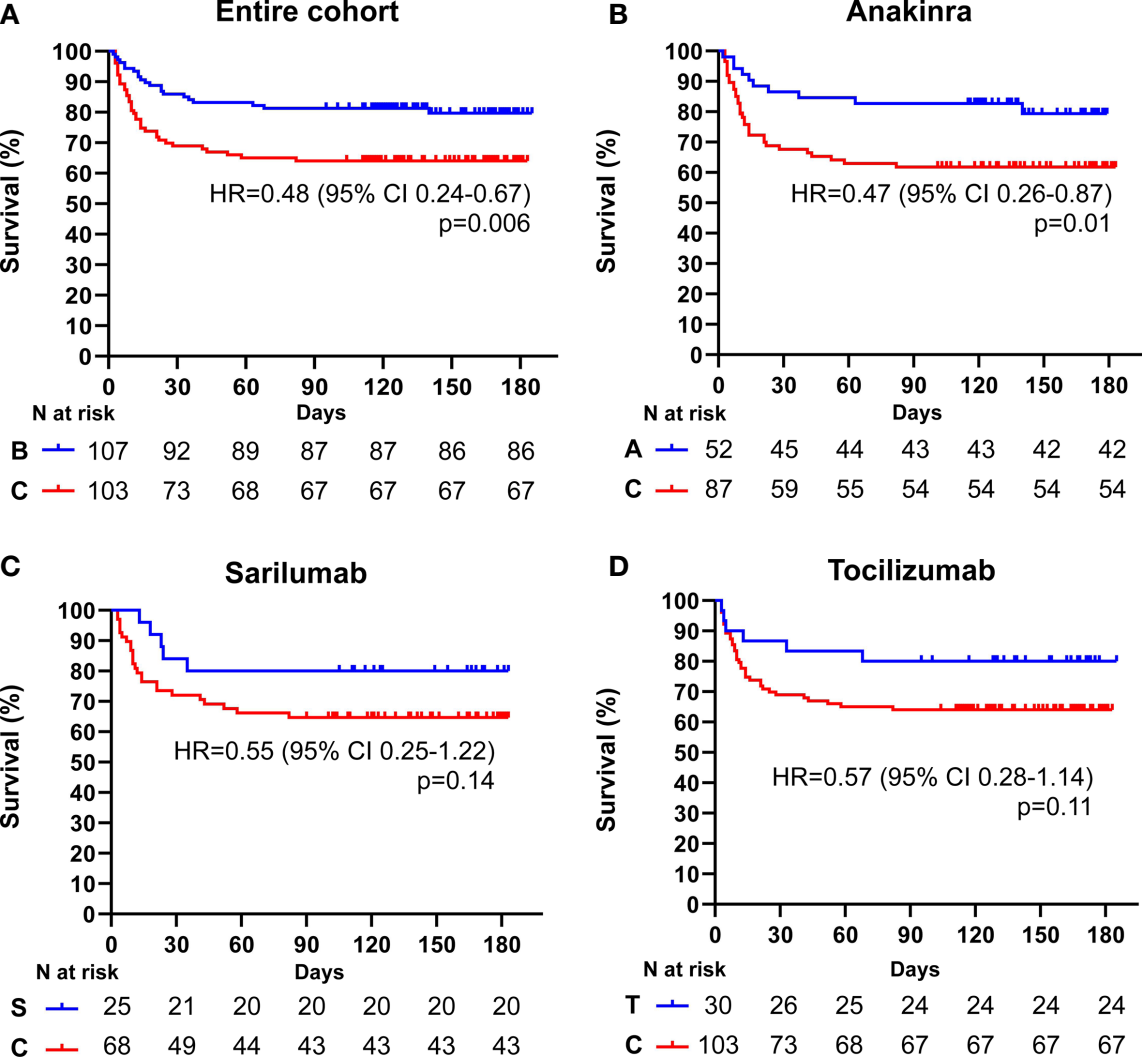
Cumulative incidence of long-term survival of patients treated with biologic drugs compared to patients treated with standard of care: entire patient cohort. Kaplan-Meier curves showing the cumulative incidence of overall survival up to 186 days of the entire cohort of patients treated with biologic drugs **(A)**, anakinra **(B)**, sarilumab **(C)**, and tocilizumab **(D)** compared to patients treated with standard of care. Time to death is expressed in days from the day of enrolment. A, anakinra; B, biologic drugs; C, comparators treated with standard of care; S, sarilumab; T, tocilizumab; HR, hazard ratio; CI, confidence interval. Statistically significant p value < 0.05.

After a median follow-up time of 111 days (range 3-186) from the fulfilment of inclusion criteria, 21 (19.6%) patients treated with biologics and 36 (34.9%) patients treated with standard of care died (p = 0.01). Treatment with biologic drugs was associated with a lower mortality risk compared to patients treated with standard of care (Hazard Ratio (HR) 0.48; 95% CI 0.29-0.81; p = 0.006) (**Figure 1A**). When the efficacy of each biologic agent was compared to matched controls treated with standard of care, anakinra (HR 0.47; 95% CI 0.26-0.87; p = 0.01), but not sarilumab (HR 0.55; 95% CI 0.25-1.22; p = 0.14) nor tocilizumab (HR 0.57; 95% CI 0.28-1.14; p = 0.11), provided a statistically significant lower mortality risk (**Figures 1B–D** and **Tables 2** **–** **4**). In the multivariate analysis, treatment with cytokine blocking agents was associated with improved patient survival while older age and high concentration of lactate dehydrogenase were independently associated with overall increased mortality (**Table 5**). When considering only patients treated with biologic therapies, COVID-19 related mortality was independently associated with older age, high concentration of lactate dehydrogenase, and low PaO2/FiO2 ratio at the time of drug infusion (**Table 6**).

**Table 5:** Baseline univariate and multivariate predictors of mortality in the patients’ cohort.

	Univariate analysis		Multivariate analysis	
	HR (95% CI)	p value	HR (95% CI)	p value
**Age**	1.05 (1.02-1.08)	**0.0001**	1.05 (1.02-1.08)	**0.001**
**Sex (F)**	0.64 (0.37-1.1)	0.11		
**HBP**	1.62 (0.97-2.71)	0.07	1.39 (0.77-2.51)	0.17
**CAD**	2.23 (1.2-4.16)	**0.011**	1.59 (0.82-2.99)	0.16
**PaO2/FiO2 ratio (mmHg)**	0.997 (0.99-1.00)	0.09	0.998 (0.99-1.002)	0.51
**LAD**	1.002 (1.001-1.003)	**0.002**	1.002 (1-1.003)	**0.003**
**CRP**	1.003 (1-1.01)	**0.05**	1.001 (0.99-1.01)	0.36
**Ferritin**	1 (1-1)	0.56		
**IL-6**	1 (1-1)	0.11		
**Treatment with biologics**	0.4 (0.23-0.68)	**0.001**	0.49 (0.27-0.87)	**0.01**

Results are reported as hazard ratio and 95% confidence interval; statistically significant p value < 0.05. HBP, high blood pressure; CAD, coronary artery disease; LAD, lactate dehydrogenase; CRP, C-reactive protein; IL-6, interleukin-6; HR, hazard ratio; CI, confidence interval. Bold values indicate that the p values are statistically significant.

**Table 6:** Baseline univariate and multivariate predictors of mortality in patients treated with biologic drugs.

	Univariate analysis		Multivariate analysis	
	HR (95% CI)	p value	HR (95% CI)	p value
**Age**	1.04 (0.99-1.08)	0.07	1.043 (1-1.09)	**0.048**
**Sex (F)**	1.039 (0.306-3.527)	0.95		
**HBP**	1.28 (0.54-3.04)	0.574	.	
**CAD**	4.89 (1.89-12.64)	**0.001**	1.95 (0.61-6.3)	0.27
**PaO2/FiO2 ratio (mmHg)**	0.98 (0.96-99)	**0.01**	0.98 (0.965-0.997)	**0.045**
**LAD**	1.004 (1.00-1.01)	**0.002**	1.004 (1.001-1.007)	**0.01**
**CRP**	1.001 (0.996-1.007)	0.61		
**Ferritin**	1 (1-1)	0.65		
**IL-6**	1 (0.99-1.01)	0.12		

Results are reported as hazard ratio and 95% confidence interval; statistically significant p value < 0.05. HBP, high blood pressure; CAD, coronary artery disease; LAD, lactate dehydrogenase; CRP, C-reactive protein; IL-6, interleukin-6; HR, hazard ratio; CI, confidence interval. Bold values indicate that the p values are statistically significant.

### Impact of Respiratory Distress on Clinical Response to Biologic Therapy

Because PaO2/FiO2 ratio represented an independent predictor of COVID-19 related mortality at multivariate analysis in patients treated with biologic agents, as well as a surrogate of disease progression and lung consolidation (4), we assessed the efficacy of anti-cytokine therapies on long-term survival in patients with moderate (PaO2/FiO2 ratio ≥ 100 mmHg) and severe (PaO2/FiO2 ratio < 100 mmHg) ARDS.

**Figure 2:**
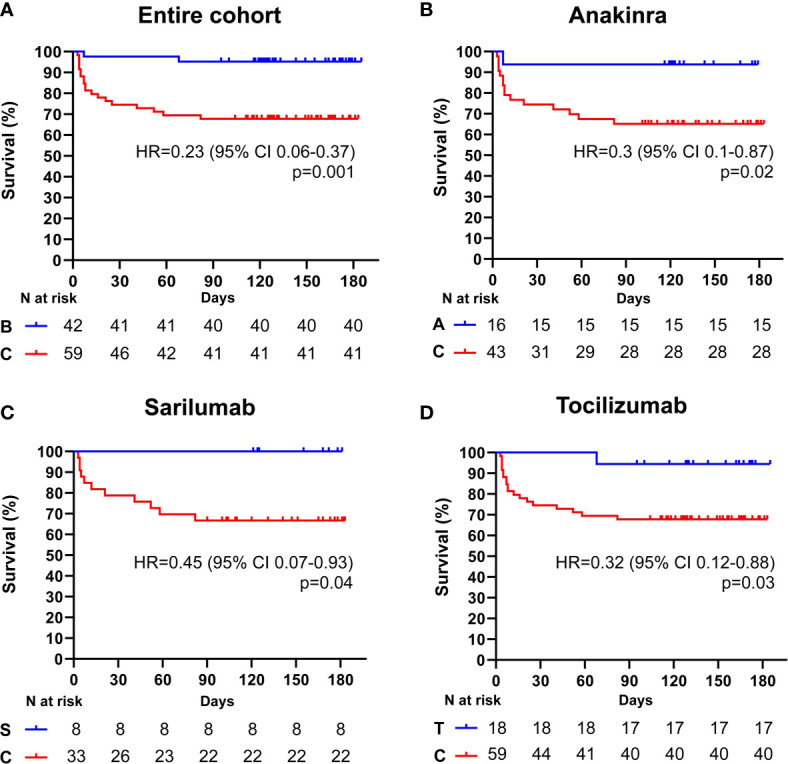
Cumulative incidence of long-term survival of patients treated with biologic drugs compared to patients treated with standard of care (PaO2/FiO2 ratio ≥ mmHg). Kaplan-Meier curves showing the cumulative incidence of overall survival up to 186 days of patients presenting with a PaO2/FiO2 ratio ≥ mmHg and treated with biologic drugs **(A)**, anakinra **(B)**, sarilumab **(C)**, and tocilizumab **(D),** compared to standard of care. Time to death is expressed in days from the day of enrolment. A, anakinra; B, biologic drugs; C, comparators treated with standard of care; S, sarilumab; T, tocilizumab; HR, hazard ratio; CI, confidence interval. Statistically significant p value < 0.05.

Among the 101 subjects with a PaO2/FiO2 ratio ≥ 100 mmHg, 42 (41.6%) received an IL-inhibitor and 59 (54%) were treated with standard of care. The two populations displayed similar epidemiological, clinical, and inflammatory parameters (**Table 7**). After a mean follow-up time of 113 days (range 95-186), 2 (4.7%) patients treated with biologic agents and 18 (30.5%) patients treated with standard of care died (p < 0.001) (HR 0.23; 95% CI 0.1-0.55; p = 0.001) (**Figure 2A**). At multivariate analysis ferritin elevation and treatment with standard of care represented independent predictors of death while treatment with anti-cytokine therapy represented a protective factor (**Table 8**). Anakinra, sarilumab, and tocilizumab were each associated with a statistically significant survival advantage compared to matched comparators (p < 0.05 for all comparisons) (**Figures 2B–D** and **Tables 2**–**4**).

**Table 7:** Clinical and serological features of patients with a PaO2/FiO2 ratio < 100 mmHg and ≥ 100 mmHg.

	PaO2/FiO2 ratio ≥ 100 mmHg			PaO2/FiO2 ratio < 100 mmHg		
	Biologics (n=42)	Standard of care (n=59)	p value	Biologics (n=65)	Standard of care (n=44)	p value
**Age (years)**	58 (51-68.25)	61 (55-71)	0.46	62 (55.5-73.5)	63 (58-73)	0.27
**Male sex, n (%)**	31 (74%)	38 (64%)	0.51	36 (55%)	19 (43%)	0.22
**Comorbidities**						
HBP	12 (29%)	22 (37%)	0.39	27 (42%)	23 (52%)	0.33
CAD	1 (2%)	10 (17%)	**0.03**	10 (15%)	8 (18%)	0.99
Type 2 diabetes	3 (7%)	10 (17%)	0.23	14 (22%)	11 (25%)	0.65
COPD	0 (0%)	3 (5%)	0.25	4 (6%)	4 (9%)	0.71
Cancer	2 (5%)	7 (12%)	0.3	3 (5%)	1 (2%)	0.65
CRF	0 (0%)	3 (5%)	0.26	7 (11%)	4 (9%)	0.99
**PaO2/FiO2 ratio (mmHg)**	144 (119-185)	172 (127-229)	0.1	77 (67-87)	73 (61-86)	0.59
**Laboratory values**						
LAD (125-220 IU/L)	432 (350-547)	415 (377-540)	0.71	487 (407-595)	565 (436-726)	0.07
CRP (< 6 mg/L)	134 (97-172)	153 (123-192)	0.07	166 (122-239)	202 (135-238)	0.41
Ferritin (30-400 ng/mL)	1422 (1054-2835)	2326 (1254-3215)	0.18	1731 (938-3099)	1717 (1198-3871)	0.28
IL-6 (< 7 pg/mL)	56 (37-113)	51 (26-116)	0.49	58 (37-120)	105 (66-286)	0.08

Results are reported as median (interquartile range) unless specified; statistically significant p value < 0.05. HBP, high blood pressure; CAD, coronary artery disease; COPD, chronic obstructive pulmonary disease; CRF, chronic renal failure; LAD, lactate dehydrogenase; CRP, C-reactive protein; IL-6, interleukin-6. Bold values indicate that the p values are statistically significant.

**Table 8:** Baseline univariate and multivariate predictors of mortality in patients with a PaO2/FiO2 ratio < 100 mmHg and ≥ 100 mmHg.

	HR (95% CI)	p value	HR (95% CI)	p value
**PaO2/FiO2 ratio** **≥ 100 mmHg**				
**Age**	1.05 (1-1.1)	**0.038**	1.05 (0.98-1.13)	0.16
**Sex (F)**	0.56 (0.23-1.39)	0.21		
**HBP**	2.32 (0.99-5.48)	0.07	1.84 (0.35-9.67)	0.47
**CAD**	1.73 (0.5-5.96)	0.39		
**LAD**	1.001 (1-1.003)	0.14		
**CRP**	1.006 (1-1.01)	**0.04**	1.004 (0.99-1.01)	0.22
**IL-6**	1 (1-1)	0.14		
**Ferritin**	1 (1-1)	**0.008**	1 (1-1)	**0.01**
**Treatment with biologics**	0.1 (0.02-0.42)	**0.002**	0.09 (0.02-0.51)	**0.006**
**PaO2/FiO2 ratio** **< 100 mmHg**				
**Age**	1.05 (1.02-1.09)	**0.004**	1.054 (1.02-1.09)	**0.003**
**Sex (F)**	0.7 (0.36-1.35)	0.29		
**HBP**	1.21 (0.63-2.3)	0.57		
**CAD**	2.37 (1.15-4.91)	**0.02**	1.63 (0.77-3.45)	0.2
**LAD**	1.002 (1-1.003)	**0.02**	1.002 (1-1.003)	**0.013**
**CRP**	1.001 (0.99-1.01)	0.57		
**IL-6**	1 (1-1)	0.37		
**Ferritin**	1 (1-1)	0.39		
**Treatment with biologics**	0.55 (0.28-1.04)	0.07	0.66 (0.33-1.33)	0.24

Results are reported as hazard ratio and 95% confidence interval; statistically significant p value < 0.05. HBP, high blood pressure; CAD, coronary artery disease; LAD, lactate dehydrogenase; CRP, C-reactive protein; IL-6, interleukin-6; HR, hazard ratio; CI, confidence interval. Bold values indicate that the p values are statistically significant.

**Figure 3:**
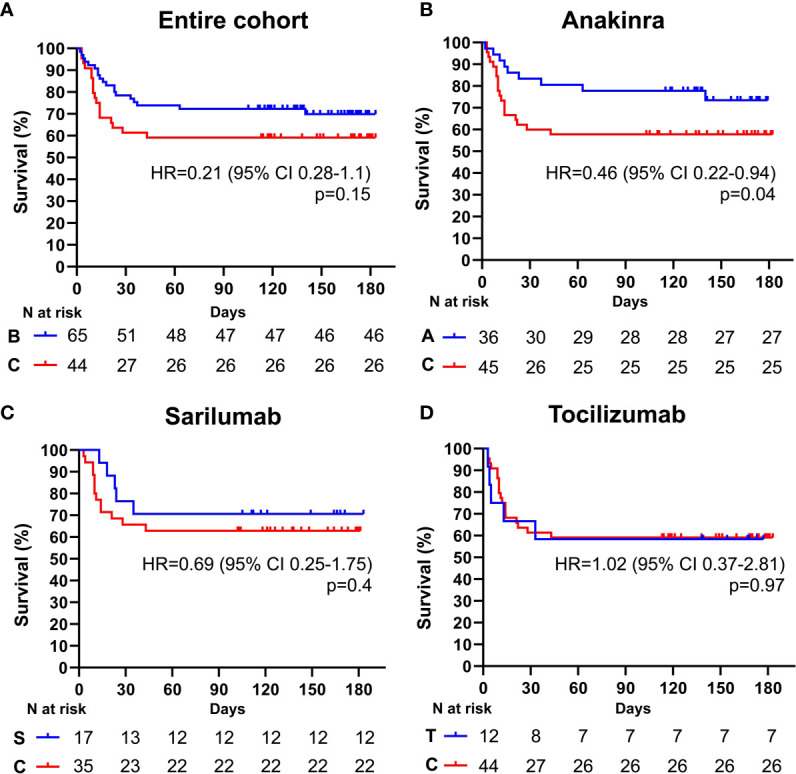
Cumulative incidence of long-term survival of patients treated with biologic drugs compared to patients treated with standard of care (PaO2/FiO2 ratio < mmHg). Kaplan-Meier curves showing the cumulative incidence of overall survival up to 186 days of patients presenting with a PaO2/FiO2 ratio < mmHg and treated with biologic drugs **(A)**, anakinra **(B)**, sarilumab **(C)**, and tocilizumab **(D)**, compared to standard of care. Time to death is expressed in days from the day of enrolment. A, anakinra; B, biologic drugs; C, comparators treated with standard of care; S, sarilumab; T, tocilizumab; HR, hazard ratio; CI, confidence interval. Statistically significant p value < 0.05.

Among the 109 patients with a PaO2/FiO2 ratio < 100 mmHg, 65 (59.6%) received an IL-inhibitor and 44 (40.4%) were treated with standard of care. The two populations displayed similar epidemiological, clinical, and inflammatory parameters (**Table 7**). After a mean follow-up time of 110 days (range 95-170), 19 (29.2%) patients treated with biologic agents and 18 (40.9%) patients treated with only local standard of care died (p = 0.21) (HR 0.61; 95% CI 0.31-1.19; p = 0.15) (**Figure 3A**). At multivariate analysis, lactate dehydrogenase and age represented independent risk factors for death (**Table 8**). In this cohort of critical patients, treatment with anakinra but not sarilumab nor tocilizumab was associated with a statistically significant lower mortality risk compared to controls (HR 0.46; 95% CI 0.22-0.94; p = 0.04) (**Figures 3B–D** and **Tables 2** **–** **4**).

## Discussion

During the first COVID-19 pandemic wave that struck Northern Italy between February and May 2020, our Hospital was at the frontline of an unprecedented health emergency (13, 14). Based on accumulating evidence about a maladaptive inflammatory response to Sars-CoV-2 in severe COVID-19 cases, we developed a set of stringent criteria to identify hyper-inflamed COVID-19 patients at risk of rapid deterioration who might have benefitted from cytokine-blocking therapies (3, 4, 9, 10, 15, 16). In the present study, all patients treated with anakinra, sarilumab, or tocilizumab were retrospectively matched according to respiratory and inflammatory parameters, and compared to a cohort of contemporary patients featuring similar characteristics treated with standard of care alone.

By analyzing clinical and serological variables associated with positive response to anti-cytokine therapies, we found that IL-1 and IL-6 inhibition improved long-term survival when initiated in the early phases of COVID-19 pneumonia before the establishment of severe ARDS (namely, PaO2/FiO2 ratio < 100 mmHg). The survival advantage offered by sarilumab and tocilizumab, in fact, was lost in patients with severe respiratory failure, and the efficacy of anakinra was markedly reduced although still proving superior to standard management. These findings demonstrate that the likelihood of response to IL-1 or IL-6 blocking strategies in patients with COVID-19 depends on the degree of respiratory impairment at the time of treatment administration and underscore the existence of a window of opportunity in which cytokine-blocking agents - as well as of more common anti-inflammatory therapies such as glucocorticoids and colchicine - might effectively counteract rampant inflammation in COVID-19 (17–19). In addition, our observations suggest that IL-6 is probably not the only driving pathway of severe COVID-19 and that molecules upstream of IL-6 in the inflammatory cascade, such as IL-1, might represent more suitable targets to quench Sars-CoV-2 induced hyper-inflammation. These results expand on our previous experience about the higher efficacy of IL-1 and IL-6 blockade in COVID-19 patients with elevated C-reactive protein and indicate that anti-cytokine treatments should be tailored not only on the inflammatory phenotype but also on the respiratory status in order to implement the selection of patients most likely to benefit from cytokine-targeted therapies (3, 20).

This work has major strengths and also limitations. In particular, results were obtained on a large and homogeneous cohort of patients that was enrolled with uniform stringent criteria and managed in the same referral center. In addition, this cohort was assembled before the reports of glucocorticoid benefits thus limiting potential treatment confounders. On the other hand, we recognize that the non-randomized retrospective observational nature of this study limits interpretation of the results and precludes their systematic application in clinical practice. Yet, randomized trials comparing the efficacy of different agents are uncommonly designed especially in the setting of a pandemic, and cohort studies, if appropriately interpreted, can allow for physician thoughtfulness and personalized medicine.

In conclusion, by unveiling a potential window of opportunity to optimize the efficacy of anti IL-1 or IL-6 inhibitors our study provides valuable information not only for interpreting available evidence from past investigations but also for designing future clinical trials.

## Data Availability Statement

The original contributions presented in the study are included in the article/supplementary material. Further inquiries can be directed to the corresponding author.

## Ethics Statement 

The studies involving human participants were reviewed and approved by COVIDBioB Study, Ethical Committee approval no. 34/int/2020, ClinicalTrials.gov NCT04318366. The patients/participants provided their written informed consent to participate in this study.

## Author Contributions

All authors contributed to the article and approved the submitted version. All authors agree to be accountable for all aspects of the work in ensuring that questions related to the accuracy or integrity of any part of the work are appropriately investigated and resolved.

## Collaborative Contributors

**BIO-RAF Study Group members:** Ada Carla Alba, Elena Baldissera, Costanza Bagnati, Cristina Barberio, Luca Benassi, Nicola Boffini, Enrica P. Bozzolo, Cecilia Bussolari, Stefania Calvisi, Corrado Campochiaro, Giuseppina Maria Casiraghi, Jacopo Castellani, Davide Catarinella, Ludovica Cavallo, Elena Cinel, Giuseppe Dalessandro, Valentina Da Prat, Adriana Cariddi, Antonella Castagna, Giulio Cavalli, Maria Pia Cicalese, Fabio Ciceri, Nicola Compagnone, Lorenzo Dagna, Francesco De Cobelli, Giacomo De Luca, Emanuel Della-Torre, Giuseppe Di Lucca, Gaetano di Terlizzi, Elisabetta Falbo, Nicola Farina, Maria Fazio, Marica Ferrante, Carola Galbiati, Gabriele Gallina, Bruno Nicolò Germinario, Giuseppe Giardina, Giovanni Gobbin, Francesca Guzzo, Giovanni Landoni, Gaetano Lombardi, Marco Lanzillotta, Nicolò Maimeri, Gaia Mancuso, Elena Moizo, Marco Montagna, Giacomo Monti, Luca Moroni, Milena Mucci, Cristina Nakhnoukh, Francesco Giuseppe Nisi, Alessandro Ortalda, Diego Palumbo, Nicola Pasculli, Chiara Pomaranzi, Marco Ripa, Patrizia Rovere-Querini, Annalisa Ruggeri, Silvia Sartorelli, Paolo Scarpellini, Tommaso Scquizzato, Alessandro Tomelleri, Moreno Tresoldi, Alberto Zangrillo.

## Conflict of Interest

The authors declare that the research was conducted in the absence of any commercial or financial relationships that could be construed as a potential conflict of interest.
